# Paediatric out-of-hospital cardiac arrest: Time to update registries?

**DOI:** 10.1186/s13054-023-04582-8

**Published:** 2023-08-02

**Authors:** Stephan Katzenschlager, Inga K. Kelpanides, Eirik Skogvoll, Guro Grindheim, Jan Wnent, Erik Popp, Markus A. Weigand, Jo Kramer-Johansen, Ingvild B. M. Tjelmeland, Jan-Thorsten Gräsner

**Affiliations:** 1 Department of Anaesthesiology, Heidelberg University Hospital, Im Neuenheimer Feld 420, 69120 Heidelberg, Germany; 2 Division of Emergencies and Critical Care, Department of Research and Development, Oslo University Hospital, Oslo, Norway; 3 Faculty of Medicine, Institute of Clinical Medicine, University of Oslo, Oslo, Norway; 4 Clinic of Anaesthesia and Intensive Care, St Olav’s Hospital, Trondheim University Hospital, Trondheim, Norway; 5 Department of Circulation and Medical Imaging, Norwegian University of Science and Technology, Trondheim, Norway; 6 Division of Emergencies and Critical Care, Department of Anaesthesiology and Intensive Care, Oslo University Hospital, Oslo, Norway; 7 Institute for Emergency Medicine, University Hospital Schleswig-Holstein, Kiel, Germany; 8 Department of Anesthesiology and Intensive Care Medicine, University Hospital Schleswig-Holstein, Campus Kiel, Kiel, Germany; 9 School of Medicine, University of Namibia, Windhoek, Namibia; 10 Division of Prehospital Services, Oslo University Hospital, Oslo, Norway

Each out-of-hospital cardiac arrest (OHCA) is a rare and dramatic event for all involved and yet requires specific interventions in a time-dependent, high-functioning chain of survival to maximise chances. Emergency medical systems (EMS) use OHCA preparedness and results to dimension and benchmark their services. International evidence-based guidelines prescribe the targets [1, 2], Utstein consensus templates define the dataset and variables [3], and OHCA registries monitor results and provide information for EMS planning and improvement.

Registries have advanced our knowledge of national and international OHCA epidemiology [4, 5], and laid foundations for further quality improvements and research. Children make up a small, albeit important, minority of the OHCA cohort. Current datasets in OHCA registries most accurately mirror the adult population.

In two population-based registry studies of children aged 0–17, the authors found similarly low incidences of cardiac arrest [6]. The studies also showed diversity in causes, contrasting with the adult population where cardiac causes predominate. In line with the previous literature, outcome was poor with high mortality rates across all age groups [7]. During data aggregation, some parameters were found to fit adults better than children. This highlights opportunities to adapt registries so as to capture a more accurate picture of paediatric cardiac arrests.

Anatomical, pathological, and psychosocial differences between adults and children influence resuscitation algorithms and outcome after cardiac arrest. To have an accurate and relevant record of paediatric cardiac arrests, we argue that a registry dataset should consider such differences. Examples of important variables specific to paediatric cardiac arrest can be found across the stages of resuscitation.

Some registries may already have implemented the proposed changes; however, by defining core variables this can be extended throughout all registries. Children are not small adults and are characterised by huge variation in size. Advanced life support (ALS) in children requires weight adjustment of medication, energy doses, and equipment. To allow for audit and quality improvement of paediatric ALS, knowledge of weight and doses of drugs and electricity given would be beneficial. These parameters can be implemented as continuous variables, ideally extracted from the electronic EMS documentation.

Pre-OHCA functional status and post-OHCA outcome assessment should employ age-adapted tools or tools validated across all age categories. Current registries, including those providing data for the authors’ recent studies, do not consistently apply specific paediatric outcome measures, such as the paediatric cerebral performance categories (PCPC) [8]. In 2020, an advisory statement from the International Liaison Committee on Resuscitation prepared by a multidisciplinary group, described a “Paediatric Core Outcome Set for Cardiac Arrest” [9]. This aimed to address the problem of inconsistent reporting of outcome in paediatric cardiac arrest, by recommending standard parameters to allow a reliable determination of functional status. In parallel with paediatric specific outcome parameters, intensive care units receiving children after OHCA should participate in their national cardiac arrest registry. In a more recent development, the Norwegian cardiac arrest registry now collects patient-reported outcome measures (PROM) from adult survivors to capture the patients’ perspective in outcome [10]. Paediatrics-adapted PROMs, for completion by children and/or caregivers, could be a means of including longer-term outcomes in registries.

In 1995, a task force consisting of members from the American Academy of Pediatrics, the American Heart Association, and the European Resuscitation Council proposed the “Paediatric Utstein Style”, a format for reporting cases of cardiac arrest in children [11]. Since then, research studies carried out have inconsistently adhered to the proposed format, and no revisions have been published. Recommended parameters have not been universally incorporated into established cardiac arrest registries. Some concepts proposed are now less used, such as return of spontaneous ventilation and differentiation between respiratory and cardiac arrest. Demographics describing emergency departments and intensive care units can be of interest for specific research questions; however, the proposed core parameters are not captured by the two bespoke registries. As a consequence, dedicated paediatric studies have been difficult to compare due to inconsistent choice of age subgroupings, treatment parameters, and outcome measures [12, 13]. In line with updated resuscitation guidelines, up-to-date reporting guidelines are therefore also needed. Such updates to recommended registry data have resource implications for registries and must balance detail against feasibility and completeness. By utilising established registries and adding important variables specifically for the paediatric population, one can envisage improving the accuracy in reporting of paediatric OHCA. As the paediatric cohort is small, collecting additional dedicated data may not substantially increase the overall workload. These adaptations can aid understanding and improve care and outcome in children after OHCA.

Whilst conducting two studies using existing registry data, we identified a need for improvement in reporting OHCA in children, through adaptations of datasets. Ideally, changes should be made based on consensus guidelines—perhaps it is time to revisit the paediatric Utstein guidance? Given the diverse aetiology of cardiac arrest in childhood, a multidisciplinary approach underpinning such guidance would be a strength. Stakeholders from relevant specialities with expertise in managing the causes of cardiac arrest, resuscitation, and post-resuscitation care, as well as long-term rehabilitation, aided by survivors and families, could provide valuable input. By more closely tailoring registries to fit the needs of the paediatric population, we can ensure children benefit from high-quality registry data to the same extent as adults.

## Data Availability

Not applicable.
